# Genetic Diversity Among *Mycobacterium avium* Subspecies Revealed by Analysis of Complete Genome Sequences

**DOI:** 10.3389/fmicb.2020.01701

**Published:** 2020-08-07

**Authors:** John P. Bannantine, Cyril Conde, Darrell O. Bayles, Maxime Branger, Franck Biet

**Affiliations:** ^1^USDA-Agricultural Research Service, National Animal Disease Center, Ames, IA, United States; ^2^INRAE, Université de Tours, ISP, Nouzilly, France

**Keywords:** genomics, RefSeq, *Mycobacterium*, pangenome, phylogeny, paratuberculosis, whole genome comparison, recombination

## Abstract

*Mycobacterium avium* comprises four subspecies that contain both human and veterinary pathogens. At the inception of this study, twenty-eight *M. avium* genomes had been annotated as RefSeq genomes, facilitating direct comparisons. These genomes represent strains from around the world and provided a unique opportunity to examine genome dynamics in this species. Each genome was confirmed to be classified correctly based on SNP genotyping, nucleotide identity and presence/absence of repetitive elements or other typing methods. The *Mycobacterium avium* subspecies *paratuberculosis* (*Map*) genome size and organization was remarkably consistent, averaging 4.8 Mb with a variance of only 29.6 kb among the 13 strains. Comparing recombination events along with the larger genome size and variance observed among *Mycobacterium avium* subspecies *avium* (*Maa*) and *Mycobacterium avium* subspecies *hominissuis* (*Mah*) strains (collectively termed non-*Map*) suggests horizontal gene transfer occurs in non-*Map*, but not in *Map* strains. Overall, *M. avium* subspecies could be divided into two major sub-divisions, with the *Map* type II (bovine strains) clustering tightly on one end of a phylogenetic spectrum and *Mah* strains clustering more loosely together on the other end. The most evolutionarily distinct *Map* strain was an ovine strain, designated Telford, which had >1,000 SNPs and showed large rearrangements compared to the bovine type II strains. The Telford strain clustered with *Maa* strains as an intermediate between *Map* type II and *Mah*. SNP analysis and genome organization analyses repeatedly demonstrated the conserved nature of *Map* versus the mosaic nature of non-*Map M. avium* strains. Finally, core and pangenomes were developed for *Map* and non-*Map* strains. A total of 80% *Map* genes belonged to the *Map* core genome, while only 40% of non-*Map* genes belonged to the non-*Map* core genome. These genomes provide a more complete and detailed comparison of these subspecies strains as well as a blueprint for how genetic diversity originated.

## Introduction

The *Mycobacterium avium* complex (MAC) comprises three species which include *M. avium*, *M. intracellulare*, and more recently, *M. chimaera* (Tortoli et al., 2004). There currently exist four subspecies within *M. avium*, which are the focus of this study. These include: *avium*, *hominissuis*, *paratuberculosis*, and *silvaticum*. The subspecies designations of *M. avium* have recently been confirmed by calculating average nucleotide identity (ANI) and genome-to-genome distance pairwise values (Tortoli et al., 2019). *M. avium* infections in humans manifest in three different forms: lymphadenitis (Christensen and Koeppe, 2010), disseminated (Ohkusu et al., 2004), or the most common, pulmonary (Inderlied et al., 1993; Tran and Han, 2014). The MAC are all closely related genetically and thus it can be difficult to distinguish which species/subspecies are the cause in human infections. However, through genome sequencing, differences have been identified and highlighted, especially for *M. avium* subspecies *paratuberculosis* (*Map*), which is the causative agent of Johne’s disease in cattle, sheep and other ruminant animals. This disease is endemic in the United States and is a problem worldwide due to the severe economic consequences to dairy cattle as well as goat and sheep industries. As more complete genome sequences accumulate, the opportunity to understand the pathological and genetic distinctions between the subspecies of *M. avium* will become more apparent.

*Map* infection of ruminant animals results in a lengthy incubation that yields a chronic inflammation of the intestine which mimics symptoms of Crohn’s disease. Studies focusing on Johne’s disease commonly use the K-10 bovine strain of *Map* since it has emerged as a widely disseminated reference strain and was used as the reference *Map* strain in this study. Originally isolated in 1990 from a dairy cow in Wisconsin, the genome sequence from this strain was the first one elucidated for *Map*. The K-10 genome sequence has now been available for 15 years (Li et al., 2005) and has been useful for diagnostics, epidemiology and pathogen biology. Two dozen more genomes of *Map* have since been reported in varying degrees of completion including bovine strains (Amin et al., 2015; Mobius et al., 2017), ovine strains in the United States and Australia (Bannantine et al., 2012; Brauning et al., 2019), camel strains (Ghosh et al., 2012), and human strains (Wynne et al., 2011; Bannantine et al., 2014).

Within *Map*, two primary strain lineages have emerged that correlate to the host they are isolated from (sheep versus cattle); although host-based lineages have been blurred as more isolates are typed from deer, bison and other hosts (Bryant et al., 2016). The type I and type III strains are generally ovine isolates and the type II strains are primarily bovine, deer, bison or human isolates. In this study, we examined 12 type II bovine isolates of *Map* and one ovine isolate, which is a type I strain (Brauning et al., 2019). No type III ovine strains are represented as no complete genome sequences are yet available. Two other subspecies are represented, which include *M. avium* subspecies *avium* (*Maa*) and *M. avium* subspecies *hominissuis* (*Mah*). However, no *M. avium* subspecies *silvaticum* complete genomes have been made publicly available to date. In this study, *Mah* and *Maa* strains are combined and termed non-*Map* strains.

With the number of *M. avium* subspecies complete genomes now available, we can look in depth at the genomic diversity among these subspecies with a much higher resolution and accuracy than was possible using DNA microarrays (Paustian et al., 2005, 2008) or partial genomic sequences (Bannantine et al., 2002). As an example, the movements of the hallmark IS900 insertion elements can be tracked among sequenced strains that could not be deciphered based solely on DNA hybridizations to microarrays and average nucleotide identities can be determined at a whole genome level. This study has revealed different numbers of the taxonomically defining insertion elements, IS900 and IS1245, and in some cases, there exist only partial copies of these mobile DNA sequences. Furthermore, gene deletions could be detected by DNA microarray provided they spanned at least one open reading frame represented on the array, but with complete genome sequences, small deletions and insertions (indels) are now readily detectable. Finally, core and pangenomes can be deciphered for each subspecies, which is less accurate with fragmented genome assemblies.

Over 90% of genome sequencing projects target microbial species. Partial sequencing of over 140 *Map* isolates worldwide has demonstrated a very stable genome in this subspecies with less than 0.5 single nucleotide polymorphisms (SNPs) per genome per year (Bryant et al., 2016). This is comparable to another stable bacterium, *Listeria monocytogenes*, which showed SNP differences of less than 6 out of 2,298 genes over 2 years (Schmid et al., 2014), but more variable than the highly stable Group A *Streptococcus* showing 0.0002 core genome SNPs per site/year (Coppens et al., 2019). In this study, we conducted a multi-strain comparison of the largest set to date of complete mycobacterial genomes from the same species. We targeted 13 complete *Map* genomes for analysis, but also included other non-*Map* genomes in the *avium* species for comparison purposes and to add evolutionary insights. We further identified the core and accessory genomes of *Map* and non-*Map* strains to show how infrequent horizontal gene transfer events occur in *Map*.

## Materials and Methods

### Genomes

At the study’s conception, all *M. avium* complete genomes were download from the NCBI RefSeq database in November 2019 (Tables 1, 2). Each strain was compared with all others enrolled in this study for determining similarity using ANI and Jaccard coefficient. ANI was calculated using the OrthoANIu algorithm (Yoon et al., 2017) and Jaccard coefficient was calculated using PanOCT software (Fouts et al., 2012).

**TABLE 1:** Complete RefSeq Map genomes available in NCBI.

**RefSeq accession**	**Strain designation**	**Host**	**BioProject**	**Assembly ver.**	**Country of origin**	**Submission date**	**Sequence ID**	**Citation**
GCF_000007865.1	K-10	Bovine	PRJNA91	ASM786v1	U. S. A.	30-Jan-04	AE016958.1	Li et al., 2005
GCF_000390085.1	MAP4	Human	PRJNA168471	ASM39008v1	U. S. A.	9-May-13	CP005928.1	Bannantine et al., 2014
GCF_000835225.1	E1	Bovine	PRJNA269152	ASM83522v1	Egypt	10-Feb-15	CP010113.1	Amin et al., 2015
GCF_000835265.1	E93	Bovine	PRJNA269152	ASM83526v1	Egypt	10-Feb-15	CP010114.1	Amin et al., 2015
GCF_001653355.1	MAP/TANUVAS/TN/India/2008	Bovine	PRJNA314834	ASM165335v1	India	27-May-16	CP015495.1	Unpublished
GCF_002211525.1	JII-1961	Bovine	PRJNA390765	ASM221152v1	Germany	30-Jun-17	CP022105.1	Mobius et al., 2017
GCF_002208705.2	FDAARGOS_305	Bovine	PRJNA231221	ASM220870v2	U. S. A.	2-Mar-18	CP022095.2	Unpublished
GCF_003713025.1	MAPK_CN7/15	Bovine	PRJNA498906	ASM371302v1	South Korea	6-Nov-18	CP033428.1	Unpublished
GCF_003713045.1	MAPK_CN9/15	Bovine	PRJNA498904	ASM371304v1	South Korea	6-Nov-18	CP033427.1	Unpublished
GCF_003815795.1	MAPK_CN4/13	Bovine	PRJNA505100	ASM381579v1	South Korea	25-Nov-18	CP033910.1	Unpublished
GCF_003815815.1	MAPK_JB16/15	Bovine	PRJNA505101	ASM381581v1	South Korea	25-Nov-18	CP033911.1	Unpublished
GCF_003816035.1	MAPK_JJ1/13	Bovine	PRJNA168471	ASM381603v1	South Korea	25-Nov-18	CP033909.1	Unpublished
GCF_003957335.1	Telford 9.2	Ovine	PRJNA505099	ASM395733v1	Australia	20-Dec-18	CP033688.1	Brauning et al., 2019

**TABLE 2:** Complete Mah and Maa genomes in NCBI.

**RefSeq accession**	**Subspecies designation**	**Strain**	**BioProject**	**Assembly ver.**	**Country of origin**	**Submission date**	**Sequence ID**	**Citation**
GCF_000014985.1	*M. avium* subsp. *hominissuis*	104	PRJNA224116	ASM1498v1	U.S.A.	20-Nov-06	CP000479.1	Uchiya et al., 2013
GCF_000829075.1	*M. avium* subsp. *hominissuis*	TH135	PRJNA224116	ASM82907v1	Japan	1-Oct-13	AP012555.1	Uchiya et al., 2013
GCF_001683455.1	*M. avium* subsp. avium	RCAD0278	PRJNA224116	ASM168345v1	China	11-Jul-16	CP016396.1	Unpublished
GCF_001865635.3	*M. avium* subsp. *hominissuis*	OCU464	PRJNA224116	ASM186563v3	Japan	3-Nov-17	CP009360.3	Unpublished
GCF_001936215.1	*M. avium* subsp. *hominissuis*	H87	PRJNA224116	ASM193621v1	U.S.A.	5-Jan-17	CP018363.1	Unpublished
GCF_003408535.1	*M. avium* subsp. *hominissuis*	MAC109	PRJNA224116	ASM340853v1	U.S.A.	20-Aug-18	CP029332.1	Matern et al., 2018
GCF_003640565.1	*M. avium* subsp. avium	HJW	PRJNA224116	ASM364056v1	China	12-Oct-18	CP028731.1	Unpublished
GCF_004345205.1	*M. avium* subsp. *hominissuis*	mc2 2500	PRJNA224116	ASM434520v1	U.S.A.	4-Mar-19	CP036220.1	Unpublished
GCF_005518035.1	*M. avium* subsp. *hominissuis*	101034	PRJNA532547	ASM551803v1	U.S.A.	10-May-19	CP040247.1	Bouso and Planet, 2019
GCF_005518055.1	*M. avium* subsp. *hominissuis*	101115	PRJNA532547	ASM551805v1	U.S.A.	10-May-19	CP040255.1	Bouso and Planet, 2019
GCF_005518015.1	*M. avium* subsp. *hominissuis*	101174	PRJNA532547	ASM551801v1	U.S.A.	10-May-19	CP040250.1	Bouso and Planet, 2019
GCF_002716965.2	*M. avium* subsp. *hominissuis*	OCU873s_P7_4s	PRJNA345414	ASM271696v2	Japan	6-Feb-19	CP018020.2	Yano et al., 2017
GCF_002716905.2	*M. avium* subsp. *hominissuis*	HP17	PRJNA336241	ASM271690v2	Japan	6-Feb-19	CP016818.2	Yano et al., 2017
GCF_002716925.2	*M. avium* subsp. *hominissuis*	OCU901s_S2_2s	PRJNA345418	ASM271692v2	Japan	6-Feb-19	CP018014.2	Yano et al., 2017
GCF_009002535.1	*M. avium* subsp. *hominissuis*	JP-H-1	PRJDB8716	ASM900253v1	Japan	5-Sep-19	AP020326.1	Unpublished
No RefSeq accession	*M. avium* subsp. *hominissuis*	MAH11	PRJNA380351		Norway	5-Feb-19	CP035744.1	Dragset et al., 2019

*MAH11 was not included the genome comparisons conducted in this study since it does not yet have a RefSeq annotation.*

### Bioinformatic Analysis

Paired-ends reads (250 bp) were simulated using ART software (v2.5.8) (Huang et al., 2012) based on the HiSeq 2500 platform for each genome. Reads were then mapped to either K-10 (*Map*) or 101115 (*Mah*, plasmids ignored) reference genomes with Burrow-Wheeler Aligner (BWA) mem (v0.7.12) (Li and Durbin, 2009). SNPs were detected with FreeBayes (v1.1.0) (Garrison and Marth, 2012). SNPs were filtered out based on three criteria (i) quality of the SNP is more than 20 (ii) read depth is more than 20 and (iii) distance between 2 SNPs must be more than 10 bp to avoid sequencing error generated by the read simulation. Each VCF file was functionally annotated with the snpEff tool (v4.3) (Cingolani et al., 2012), then converted into a sqlite3 database with vcflib^1^ (vcf2sqlite3.py) and finally merged in a unique sqlite3 database for further analysis using an in-house script (tables and figures). In parallel, each VCF file was merged into a single VCF file using vcflib to build the SNP concatenate used to infer UPGMA phylogenetic trees with Bionumerics version 7.6.3 created by Applied Maths NV and available from http://www.applied-maths.com for each reference. Genomic feature comparisons were performed in MacVector 17.0.9 using the compare genomes tool. Synteny alignments were determined by Mauve. In order to avoid false indications of inversions or other rearrangements, these genomes were first aligned to start at the *dnaA* gene prior to Mauve analysis.

### Pangenome Analysis

The gbk files of each strain were retrieved from the NCBI RefSeq database and converted to gff3 format using bp_genbank2gff3. pl perl script from BioPerl library. Roary software (v3.11.2) (Page et al., 2015) was used to define the pangenome, core genome and accessory genome of each *Map* strain and non-*Map* strain separately. Roary was launched with –e and –n options to compute rapid core gene alignment. Phylogenies based on accessory genome matrix (presence/absence of gene) were generated using FastTree (v2.1) on *Map* and non-*Map* (Price et al., 2010). Roary outputs can be visualized with Phandango (Hadfield et al., 2017) and R scripts were used to view the resulting outputs. Pangenomes were also analyzed using PanOCT (Fouts et al., 2012) in conjunction with the JCVI pipeline and PanACEA visualization tool (Inman et al., 2019). PanOCT and Roary are both especially useful for clustering genes from closely related species/strains.

### Phylogenetic Analysis

Roary core gene multiple alignment in fasta format was converted to phylip format and used to infer the maximum likelihood SNP core gene phylogeny with RAxML (v8.2.11) (Stamatakis, 2014) with parameters –f a –x 123456 –p 123456 -# autoMRE –m GTRGAMMA and automatic bootstrapping. The phylogenetic tree branch lengths were readjusted for recombination sites using ClonalFrameML v1.12 (Didelot and Wilson, 2015). We also performed a split network phylogeny using Neighbor-Net analysis in SplitsTree5 v5.0.0_alpha (Bryant et al., 2007).

### Nonsynonymous-Synonymous Ratio (dN/dS) Calculation

The nucleotide and protein sequences of all core genes identified by Roary analysis were extracted and binned into two groups corresponding to *Map* and non-*Map* strains. Each protein coding sequence was aligned with MAFFT (Katoh and Standley, 2013) and converted back into nucleotide codon alignments with Pal2Nal (Suyama et al., 2006). Phylogenetic trees were inferred with FastTree 2.1.11, based on resulting nucleotide codon alignment. Both tree and nucleotide codon alignment were fed into HyPhy software using a MEME (Multiple EM For Motif Elicitation) algorithm (Murrell et al., 2012) to determine dN/dS value for each gene in each group. Grouped dN/dS values were then calculated after removing 877 recombinant genes. Finally, the dN/dS values of the remaining 1559 genes were filtered to keep only values less than 10, above which are considered artefactual (Mastrorilli et al., 2018).

### *In silico* Analysis of the *hsp65* Gene

Gene sequences were extracted from each genome and analyzed for defining SNPs. The hsp65 codes were assigned based on the nomenclature developed by Turenne and coworkers (Turenne et al., 2006).

### *In silico* MLVA Typing

MISTReSS software^2^ was used to identify in-silico MLVA profiles on complete genomes and INMV was deduced for each MLVA profile using MAC-INMV-SSR database (v3.0) (Cochard et al., 2019).

## Results

### RefSeq Genomes Using PGAP Annotation Reveals Additional Genes

We used NCBI’s RefSeq annotation for all *M. avium* genomes in this study. The *Map* bovine strain K-10 genome was initially annotated using Artemis and Glimmer in 2005 (Li et al., 2005) and most recently re-annotated using NCBI’s prokaryotic genome annotation pipeline (PGAP) (Haft et al., 2018). Hence the locus tags have now been changed from what was reported earlier in the scientific literature. To alleviate confusion and enable easy linkage of new data to publications reporting old locus tags, a supplementary table was constructed that correlates the old locus tag to the new RefSeq gene ID and corresponding protein ID (Supplementary Table S1).

A RefSeq annotation has much improved and consistent annotation across all genomes while also implementing certain quality standards, including lack of sequence contamination (Haft et al., 2018). From the K-10 RefSeq annotation, 227 newly discovered genes not present in the initial annotation were revealed and 67% of these “new” genes are annotated as hypothetical proteins (Supplementary Table S2). These genes could encode new antigens or novel virulence and metabolic functions. Conversely, 199 coding sequences have been annotated as pseudogenes (Supplementary Table S3). To be included in this study, all genome assemblies had to be complete, closed, and annotated using PGAP with a RefSeq accession number. These criteria excluded one complete genome, the *Mah* strain MAH11, which did not have a RefSeq accession when this study was conceived and has recently been published (Dragset et al., 2019). Therefore, a total of 13 *Map* genomes and 15 non-*Map* RefSeq genomes were analyzed (Tables 1, 2).

### Characteristics of *M. avium* Genomes

These *M. avium* genomes are from strains distributed across five continents: Africa, North America, Europe, Asia and Oceania. Eleven of the thirteen *Map* are bovine isolates with one human (MAP4) and one ovine strain (Telford 9.2) completing the set (Table 1). However, the human isolate groups with the bovine isolates to yield 12 type-II strains and a single type-I ovine strain. Accession, country of origin and publication information are listed in Tables 1, 2 for all of the *M. avium* subspecies genomes. *Map* genome sizes are relatively consistent despite their broad geographical distribution. An average size of 4.83 Mb + 29.6 kb was observed for *Map* while *Maa* averaged 4.96 Mb + 5.8 kb among its two genomes (Table 3). *Mah* strains had the largest average genome size at 5.25 Mb + 195.9 kb. All *Map* and 11 of the non-*Map* genomes have two non-coding RNAs, the RNase_P_RNA and SRP_RNA, 46 transfer RNAs and 3 ribosomal RNA (rRNA) genes. While three non-*Map* genomes have 47 tRNAs and one has four rRNAs (Table 3). *Map* pseudogenes range in number from 173 to 384, while non-*Map* pseudogenes range from 112 to 430. The IS900 insertion sequence element is present uniquely in *Map* (Semret et al., 2006; Singh et al., 2010; Sidoti et al., 2011) and this diagnostic element varies from 16 to 22 copies per genome. Interestingly, the IS1245 element has been considered a defining sequence for non-*Map M. avium* often used as the target in RFLP analysis (Van Soolingen et al., 1998; Johansen et al., 2005; Thibault et al., 2007), however, both *Maa* strains and one *Mah* strain do not contain this element (Table 3). Functional copies of this element vary from 0 to 36, but some strains include many additional partial copies of this insertion sequence annotated as pseudogenes (Supplementary Table S4). No *Map* or *Maa* strains possess plasmid DNA, while, eight *Mah* strains have plasmids (Table 3). The variation in genome size, gene, and pseudogene numbers suggest important differences in gene gain and loss events have occurred during evolution of the *M. avium* subspecies. One characteristic that is very consistent among all *M. avium* genomes is the uniquely high GC content (69%; Table 3).

**TABLE 3:** Characteristics of *M. avium* complex genomes in this study.

**Sequence ID**	**Subspecies designation**	**Strain**	**Genome size (bp)**	**GC content**	**Number of unique genes**	**Number of coding genes**	**Number of pseudogenes**	**tRNA**	**rRNA**	**% Hypothetical proteins**	**No. of plasmids**	**MLVA**	**INMV**	**IS900/IS1245 copies**	**hsp65 sequvar code**
AE016958.1	*paratuberculosis*	K-10	4,829,781	69.3	4,562	4,311	199	46	3	18.50	0	3-2-3-3-2-2-2-8	INMV 2	17	5
CP005928.1	*paratuberculosis*	MAP4	4,829,424	69.3	4,552	4,327	173	46	3	18.45	0	3-2-3-3-2-2-2-8	INMV 2	16	5
CP010113.1	*paratuberculosis*	E1	4,781,002	69.3	4,616	4,180	384	46	3	17.37	0	3-2-3-3-2-2-2-8	INMV 2		5
CP010114.1	*paratuberculosis*	E93	4,786,065	69.3	4,559	4,223	284	46	3	19.85	0	3-2-3-3-2-2-2-8	INMV 2		5
CP015495.1	*paratuberculosis*	MAP/TANUVAS/TN/India/2008	4,829,781	69.3	4,553	4,300	201	46	3	19.28	0	3-2-3-3-2-2-2-8	INMV 2	17	5
CP022105.1	*paratuberculosis*	JII-1961	4,829,728	69.3	4,563	4,325	186	46	3	18.47	0	3-2-3-3-2-1-2-8	INMV 6	17	5
CP022095.2	*paratuberculosis*	FDAARGOS_305	4,832,477	69.3	4,586	4,331	203	46	3	17.77	0	3-2-3-3-2-2-2-8	INMV 2	17	5
CP033428.1	*paratuberculosis*	MAPK_CN7/15	4,837,149	69.3	4,593	4,321	220	46	3	17.05	0	3-2-3-3-2-2-2-8	INMV 2	16	5
CP033427.1	*paratuberculosis*	MAPK_CN9/15	4,831,261	69.3	4,586	4,330	204	46	3	17.03	0	3-2-3-3-2-2-2-8	INMV 2	16	5
CP033910.1	*paratuberculosis*	MAPK_CN4/13	4,836,546	69.3	4,610	4,350	208	46	3	17.48	0	2-2-5-3-2-2-2-8	INMV 68	16	5
CP033911.1	*paratuberculosis*	MAPK_JB16/15	4,838,766	69.3	4,609	4,355	202	46	3	17.40	0	2-2-5-3-2-2-2-8	INMV 68	17	5
CP033909.1	*paratuberculosis*	MAPK_JJ1/13	4,838,649	69.3	4,610	4,355	203	46	3	17.38	0	2-2-4-3-2-2-2-8	INMV 149	17	5
CP033688.1	*paratuberculosis*	Telford 9.2	4,907,428	69.2	4,700	4,400	248	46	3	16.72	0	4-1-3-3-1-1.5-1-8	INMV 219	22	6
CP000479.1	*hominissuis*	104	5,475,491	69.0	5,199	4,894	248	46	3	18.16	0	2-5-2-2-1-1-2-9	INMV 18	25	1
AP012555.1	*hominissuis*	TH135	4,951,217	69.3	4,633	4,476	112	46	3	16.94	1	1-2-4-3-NA-1.5-3-8		1	9
CP016396.1	avium	RCAD0278	4,953,610	69.3	4,647	4,435	139	46	3	16.68	0	2-4-1-3-1-1-2-7	INMV 100	0	4
CP009360.3	*hominissuis*	OCU464	5,178,230	69.1	4,917	4,708	164	47	3	15.25	2	1-1-3-3-NA-1-2-8		0	2
CP018363.1	*hominissuis*	H87	5,626,623	68.8	5,276	4,955	268	47	3	16.87	0	3-3-3-3-1-1-5-8	INMV 206	36	2
CP029332.1	*hominissuis*	MAC109	5,188,883	69.1	4,918	4,685	192	46	4	16.35	2	0-5-3-2-1-1-5-8	New	18	1
CP028731.1	avium	HJW	4,961,843	69.3	4,683	4,454	177	46	3	15.46	0	2-3-1-3-1-1-2-7	INMV 67	0	4
CP036220.1	*hominissuis*	mc2 2500	5,438,093	68.9	5,145	4,836	264	46	3	15.92	2	0-3-3-3-NA-1-5-8		16	ND
CP040247.1	*hominissuis*	101034	5,301,832	69.0	5,032	4,679	316	46	3	15.84	2	3-5-3-3-NA-1-5-8		1	2
CP040255.1	*hominissuis*	101115	5,254,673	69.0	4,970	4,632	331	46	3	15.94	4	3-5-3-3-NA-1-5-8		1	2
CP040250.1	*hominissuis*	101174	5,101,624	69.2	4,841	4,529	271	46	3	15.37	2	3-5-3-3-NA-1-5-8		1	2
CP018020.2	*hominissuis*	OCU873s _P7_4s	5,027,323	69.2	4,739	4,563	124	46	3	15.55	0	2-1-4-3-NA-1-3-7		7	1
CP016818.2	*hominissuis*	HP17	5,100,690	69.2	4,812	4,623	137	46	3	15.77	0	1-1-4-3-NA-1.5-3-7		3	1
CP018014.2	*hominissuis*	OCU901s _S2_2s	5,186,801	69.1	4,893	4,690	151	46	3	15.92	0	1-1-4-3-NA-1.5-3-7		1	1
AP020326.1	*hominissuis*	JP-H-1	5,491,452	68.9	5,175	4,713	430	47	3	15.13	3	3-3-3-3-NA-1-5-8		4	7
CP035744.1	*hominissuis*	MAH11	5,098,805	69.2											

*The IS900/IS1245 column represents the total number of copies of IS900 present in all Map strains and total number of IS1245 in non-Map strains. Only complete copies of these elements are listed in this table. Refer to Supplementary Table S4 for a list of partial copies of IS1245. ND means not designated using Turenne et al. (2006) nomenclature. Hence it represents a new sequevar. Only chromosomally located genes are included in the gene counts for this table. No complete copies of IS900 were detected in the E1 or E93 sequence. Refer to the discussion for details. NA indicates an ambiguity in the VNTR3 loci.*

Several previously published analyses have been applied in this study to validate these genomes as *Map* or non-*Map*. For example, only *Map* strains possess a C-to-A SNP in MAP_1025, a gene encoding a proline rich protein (Bannantine et al., 2011). This defining SNP remains true among these *M. avium* strains with all 13 *Map* genomes possessing an adenine nucleotide at position 83 in MAP_1025 while all non-*Map* genomes have a cytosine at that position (Supplementary Table S5). The *hsp65* gene (aka *groEL2*, MAP_3936, and MAP_RS20190 in K-10), which encodes a heat shock protein, has been used to distinguish members of the MAC based on sequence variants at 19 positions within the gene (Turenne et al., 2006). A total of 14 different sequevars were identified among all 73 MAC isolates tested in that study (Turenne et al., 2006). All *Map* bovine strains in the current study belong to sequevar code 5 whereas the *Map* ovine strain Telford is sequevar code 6 (Supplementary Table S6). This division of bovine and ovine strains into codes 5 and 6 is consistent with what was reported by Turenne and coworkers (Turenne et al., 2006). The *Mah* genomes grouped into sequevar codes 1, 2, 7, and 9 while the two *Maa* strains were in code 4. *Mah* strain mc2 2500 was not in a sequevar previously reported. By analyzing genomes in the current study, three additional SNPs were detected in the 1,626 bp *hsp65* gene that were not reported by Turenne et al. (2006). The locations of these SNPs are highlighted in red in Supplementary Table S6.

Several large sequence polymorphism regions reported by Semret et al. (2004, 2005) are able to distinguish *Map* from non-*Map* strains. For example, the LSP^A^8 sequence is present in all non-*Map* genomes, but missing in all *Map* genomes while LSP^P^12 is present in all *Map* strains and absent from all non-*Map* strains in this study (Semret et al., 2005). Finally, the Telford strain has characterized deletions reported in all ovine *Map* strains analyzed thus far, including a 19,930 and 8,049 bp deletion relative to *Map* bovine strains (Marsh et al., 2006).

The multi-locus variable number tandem repeat analysis (MLVA) is another molecular epidemiological tool that has been standardized for MAC strains (Cochard et al., 2019). Although this tool has been outperformed by whole genome SNP analysis due to homoplasy in MLVA (Bryant et al., 2016), it is nonetheless easy to perform and has a well-established database to compare strains against. This technique, applied to the set of genomes in this study, reveals that two *Map* genomes from the South Korea strains (MAPK_CN4/13 and MAPK_JB16/15) have an INMV type code belonging to *Maa* rather than *Map*. This is code 68 (INMV type 22532228) shown in Table 3. Still another *Map* genome from the South Korea strains (MAPK_JJ1/13) has an INMV code belonging to *Mah* (code 149, INMV type 22432228). Overall, INMV2 was predominant in this collection of genomes with 7 of the 13 *Map* genomes falling in this category (Table 3). INMV codes could not be assigned to several non-*Map* strains to due ambiguities at one loci (VNTR3). These results suggest MLVA is not ideal for distinguish MAC subspecies.

### *M. avium* Genomic Diversity

**FIGURE 1:**
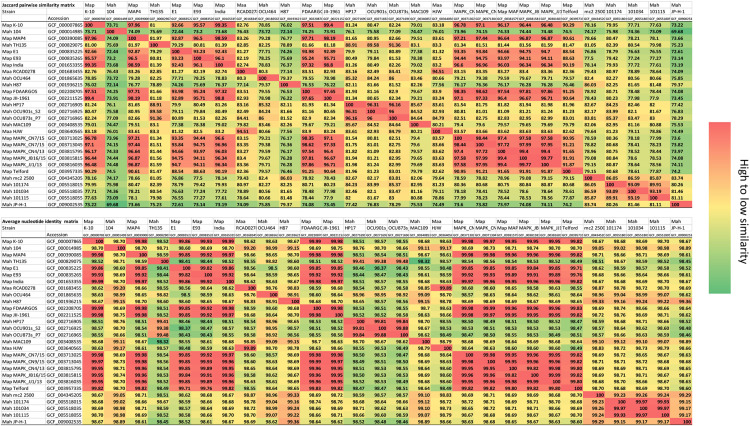
Nucleotide level similarity analysis. The top matrix shows the Jaccard pairwise cluster similarity and the bottom matrix show the average nucleotide identity (ANI). The ANI values are a similarity index between two genomes expressed as a percentage. Jaccard percentages were obtained by direct pairwise genome comparisons using PanOCT as described in the methods. Note that the Jaccard similarity has a much broader range of values. The strain names and corresponding RefSeq accession numbers are shown in both matrices. Heat map shows high similarity in red versus lower values in green.

With multiple complete genomes, investigators can now examine in more detail the genome distances among *M. avium* strains. These measurements can be obtained a variety of ways at the nucleotide level including Jaccard cluster similarity (Jay et al., 2012) and more commonly, ANI (Kim et al., 2014). ANI values showed a narrow range from 98.51 to 99.99 among all *M. avium* subspecies. This metric is used to define species and subspecies boundaries and is a better measure of relatedness than data obtained from a single gene, such as 16S rRNA. For taxonomic speciation, the ANI cutoff is historically 96% for a species (Ciufo et al., 2018) and greater than 98% for subspecies. All of the subspecies of *M. avium* are well above 98% ANI and confirm their taxonomic grouping (Figure 1). By way of comparison, *M. intracellulare* ATCC 13950 (CP003322), which is another species within the MAC, had an 86.33% ANI with *Map* FDAARGOS. The same *Map* strain shares a 79.42% ANI with the more distantly related *M. bovis* Danish 1331 (NZ_CP039850) and 63.66% when compared to *Escherichia coli* K-12 (NC_000913.3). The Jaccard similarity coefficient was more discriminating with all *M. avium* genomes in this study sharing >69.68% Jaccard pairwise similarity to each other (Figure 1). Among the *Map* strains the Jaccard similarity is above 90% except when the E1 and E93 strains from Egypt were compared with the Australian sheep strain Telford (88.5 and 88.6%, respectively). Interestingly, when comparing the non-*Map* strains against each other, the Jaccard percentages (range = 69.68–96.31) eclipsed percentages for non-*Map* to *Map* comparisons (Range = 72.44–83.9). Based on Jaccard similarity, *Mah* strain 104 is most distantly related to all other *M. avium* strains (Figure 1). These values further illustrate the diversity that exists among the non-*Map* strains.

### SNPs Among *M. avium* Subspecies

**FIGURE 2:**
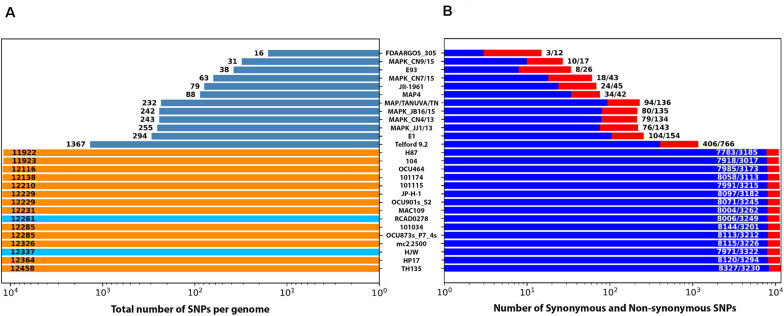
Number of single nucleotide polymorphisms among all 28 *M. avium* subspecies genomes using *Map* K-10 as the reference genome. Shown are the total number of SNPs detected in each strain **(A)** with the slate bars representing *Map*, beige bars representing *Mah* and light blue bars representing *Maa* strains. **(B)** The number of synonymous (blue bars, first value) and non-synonymous (red bars, second value) SNPs. Both graphs were plotted on a log scale with the strain designation listed in the center. Numerical differences between total SNPs and the combined nonsynonymous plus synonymous SNPs represent the intergenic SNPs.

SNPs are valuable for constructing phylogenies of closely related genomes. SNP-based analysis among the 12 *Map* type II genomes, using K-10 as the reference, shows less than 300 single nucleotide variants among them, and just over 1,000 SNPs when compared to the *Map* type I isolate (Telford 9.2; Figure 2A). When the SNPs were divided into synonymous and non-synonymous categories, there were always more amino acid-changing non-synonymous SNPs than synonymous SNPs among *Map* strains, while the opposite is true among the non-*Map* strains (Figure 2B). This suggests that there is more selective pressure on *Map*. To test this, dN/dS ratios were calculated, which is the ratio of nonsynonymous substitutions per non-synonymous site (dN) to the number of synonymous substitutions per synonymous site (dS). This ratio is used as an indicator of selective pressure acting on protein coding genes. Non-*Map* strains had a dN/dS ratio of 0.10, which shows that these strains are under a stabilizing selective pressure. In contrast, the *Map* strain dN/dS ratio is 1.27, which clearly shows the positive selective pressure these strains exhibit.

**FIGURE 3:**
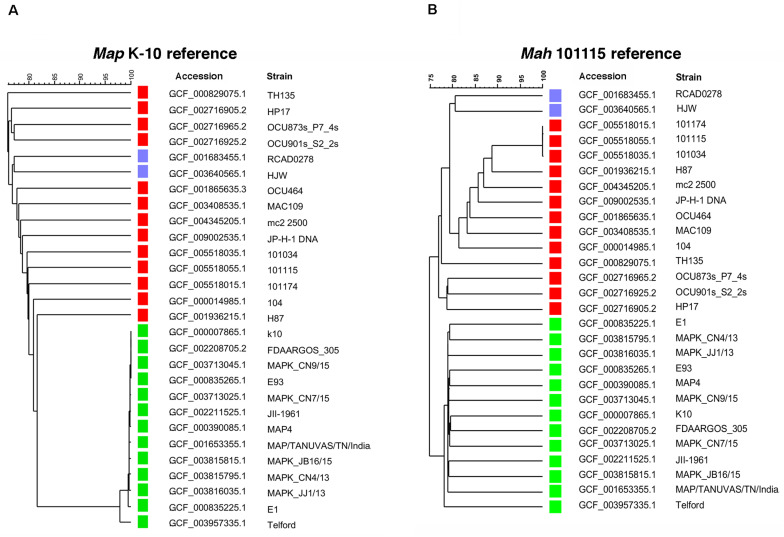
UPGMA phylogenetic tree based on the total 65401 single nucleotide variants detected in all *M. avium* strains using *Map* K-10 as the reference **(A)** or based on the total 69324 SNPs detected using *Mah* 101115 as a reference **(B)**. The green squares represent *Map* stains while red and blue represent *Mah* and *Maa* strains, respectively. Scale at the top represents percent identity.

The little *Map* diversity that does exist is not related to geographical boundaries as multiple strains from Egypt and South Korea are present in distinct branches of the phylogenetic tree (Figure 3). This result agrees with that observed by Bryant et al., which showed no association between strain relatedness with geographic location for over 140 *Map* isolates (Bryant et al., 2016). The K-10 strain is most closely related to FDAARGOS_305 and the MAPK_CN9/15 strain from South Korea. Conversely, among the *Map* bovine strains, K-10 is most distantly related to the E1 strain from Eygpt and all the bovine strains are well separated from the *Map* type I strain (Telford 9.2). Overall, even the most divergent of *Map* strains had a SNP density of only 0.284 SNPs per 1 kb. This compared to 10 SNPs per 1 kb between *Mah* 104 and MAH11 (Dragset et al., 2019). This analysis confirms that single nucleotide variants are more abundant in *Mah* than other *M. avium* subspecies (Uchiya et al., 2017). Finally, there are well over 11,500 SNPs in *Mah* and *Maa* isolates when compared to K-10 (Figure 2B) and each subspecies clustered together regardless of which strain was used as the reference (Figure 3).

### Genomic Synteny

**FIGURE 4:**
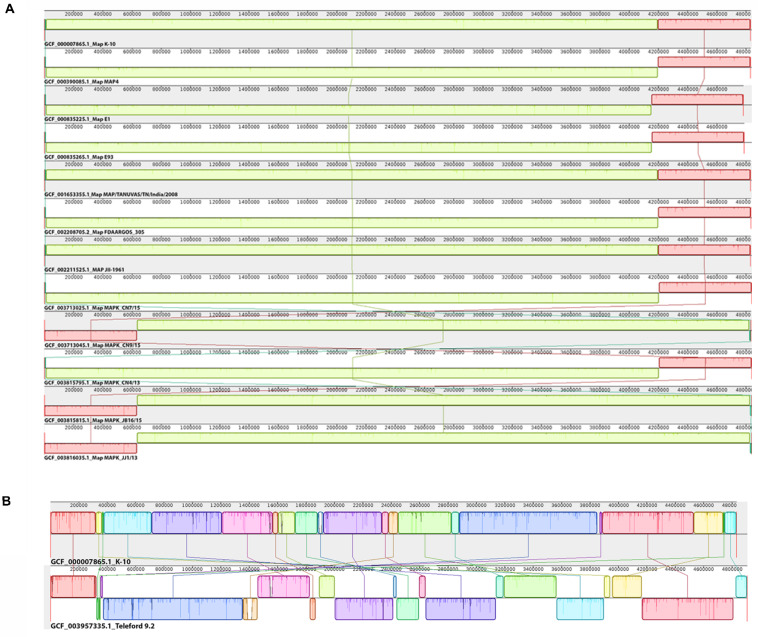
Genomic alignment between *Map* strains. The 12 type II genomes **(A)** and the type I Telford and type II K-10 genomes **(B)** were all aligned relative to the *dnaA* gene as the anchor point. The Mauve alignment is organized into one horizontal section per input genome sequence. Each genome track contains the accession number and strain of the genome sequence along with a base pair scale showing the sequence coordinates for that genome. Blocks of various colors above and beneath the center line represent defined genome regions. Similar colored blocks in different genomes represent regions that aligned in each genome. When a block lies above the center line the aligned region is in the forward orientation relative to the K-10 genome sequence (top). Blocks below the center line indicate regions that align in the reverse complement (inverse) orientation.

Genome organization among the 12 *Map* type II strains is remarkably stable with only one large inversion shared among six of the strains (Figure 4A). If the genomes were entered into Mauve without first aligning them at *dnaA*, many false rearrangements appeared (Supplementary Figure S1). The single inversion that is observed in the type II strains (Figure 4A) might be due to a mis-assembly in K-10 (Wynne et al., 2010) rather than a true DNA rearrangement. In contrast, the type I strain shows a more extensive degree of large-scale rearrangements relative to the *Map* K-10 strain despite using *dnaA* as the same starting point for alignment (Figure 4B). A genomic inversion in the center of these sequences is a primary cause of this genomic discontinuity.

### Pangenome of *M. avium* Subspecies

**FIGURE 5:**
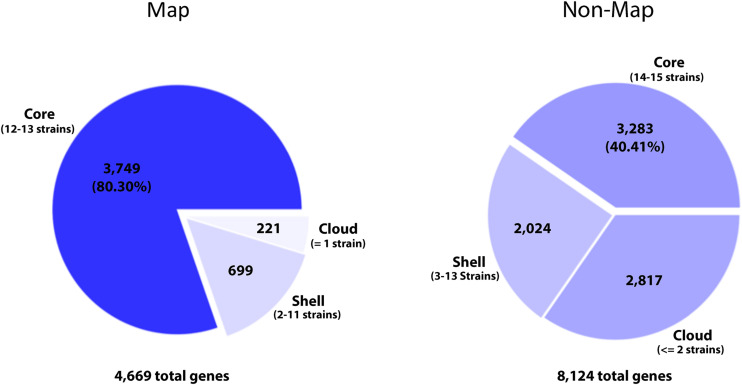
Pie chart of the pangenomes for *Map* and non-*Map* strains. Beneath each chart are the total number of genes in the pangenome. The pangenome is subdivided into core and accessory genomes. The accessory genome is further subdivided into cloud and shell genomes, which are based on the number of genomes that contain the gene(s). Also shown are the number of genes present in each category. The darker the shade of blue on the chart, the higher percentage of genes present in that category. The number of strains necessary for a gene to meet the defined category is shown in parentheses.

A significant part of the genome evolutionary process involves the extensive gain and loss of genes (Iranzo et al., 2019). Pangenome analysis should ideally identify all orthologs and distinguish them from paralogs. Analysis using Roary (with a 95% BLASTp identity cut off) identified genes present across all *Map* strains totaling 3,749, which comprises the core genome. Conversely, the accessory genome, which is subdivided into the cloud and shell genomes, total only 920 genes (Figure 5). An alternative method, PanOCT analysis, yielded a *Map* core genome comprising 3,772 genes, which is similar to the 3,749 core *Map* genes identified by Roary analysis. The pangenome of 15 non-*Map* strains comprise a total of 8,124 genes with the core genome consisting of 3,283 genes. Typically, the smaller the number of genomes analyzed, the larger the core. However, despite the similar numbers of *Map* and non-*Map* genomes in this study, there is a significantly larger core and smaller accessory genome associated with *Map* than for non-*Map* (Figure 5), suggesting a lack of horizontal gene transfer in *Map*. The *Map* core genome comprised 80% of the total genes compared to non-*Map*, which contained only 40% (Figure 5). Conversely, there were nearly 5 times more accessory genes among the non-*Map* genomes (920 *Map* versus 4,841 non-*Map* genes).

**FIGURE 6:**
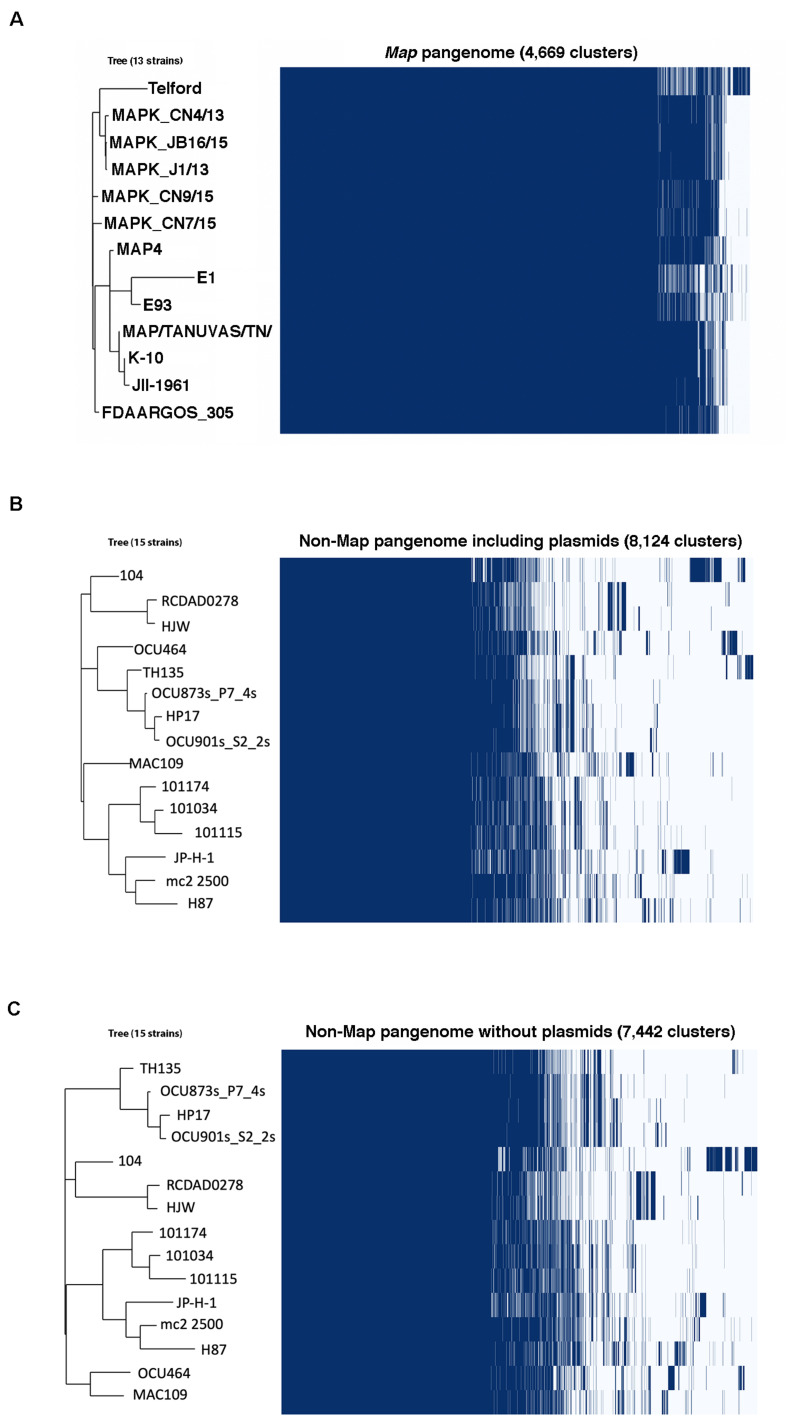
Gene presence matrix of the *Map* **(A)** and non-*Map* pangenomes analyzed with **(B)** and without **(C)** plasmid DNA elements. At the right of each panel is a compressed gene cluster matrix where blue blocks indicate presence of the gene in that cluster and white indicates its absence. Each column represents an orthologous gene family. The corresponding phylogenetic tree is presented on the left and strains listed on the tree correspond to each row of the matrix. Only the phylogeny of OCU464 is affected by the presence/absence of plasmid DNAs among the non-*Map* strains (compare **B** and **C**). Note the discrepancy of the core genome between *Map* and non-*Map*. Fully 80% of the *Map* genome consists of core genes.

In comparison to *Map*, the non-*Map* pangenome has substantially more total genes (Figures 5, 6). This is in part due to plasmid DNAs, which are not present in all strains and comprise at least a portion of the accessory genome. To determine if the presence of plasmid DNA impacted phylogenetic lineages in non-*Map* strains, Roary analysis was conducted in both circumstances. The phylogenies are very similar whether or not plasmids are included in this analysis. The only difference observed is with *Mah* OCU464, a strain which contains two plasmids (Table 3). This strain clusters with *Mah* TH135 when plasmids are included in the analysis and clusters with *Mah* MAC109 when plasmids are excluded (Figure 6). All of the gene clusters comprising the core genomes of *Map* and non-*Map* strains are listed in Supplementary Tables S7, S8. When using PanOCT, core gene clusters were, similarly, assigned (Supplementary Table S9). The pangenome map of all 28 *M. avium* strains combined is shown in Supplementary Figure S2. Collectively, the core genome of *Map* was twice the size of the core genome of non-*Map* despite having significantly fewer gene clusters and the accessory genome is very small (Figure 6). This suggests a more closed genome in *Map* with less horizontal gene transfer than non-*Map*.

**FIGURE 7:**
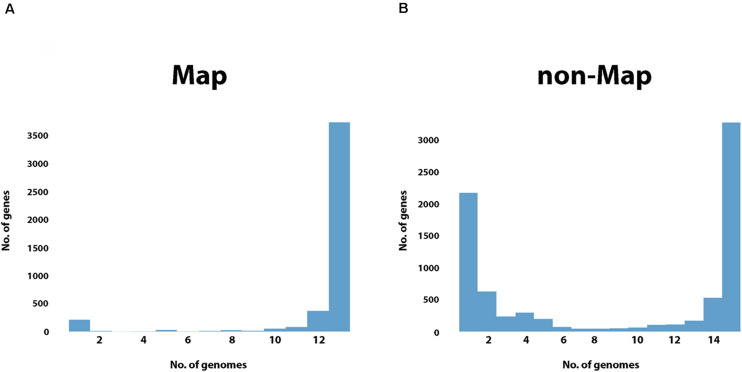
Gene frequency within *Map* or non-*Map* genomes. Shown is a histogram plot with all the genes of the *Map* **(A)** or non-*Map* **(B)** pangenomes binned by their presence in 1 or more genomes (*x*-axis). Note that most *Map* genes are present in 12 or all 13 genomes while the gene frequency in non-*Map* shows a biphasic distribution with many genes present in either a few (1–2) genomes or in most (14–15) genomes.

When quantifying the frequency of each gene in the pangenome among all the *Map* or non-*Map* genomes, a unique pattern emerges within each group (Figure 7). The *Map* genes are present in most of the genomes (12–13) while the non-*Map* genes show a more biphasic pattern where they are either in a few (1–2) genomes or in most (14–15) genomes. In contrast, very few genes are present in approximately half of the genomes regardless of subspecies (Figure 7). These data further suggest that *Map* is a closed genome while non-*Map* show evidence of horizontal gene transfer.

### Core Gene Phylogeny

**FIGURE 8:**
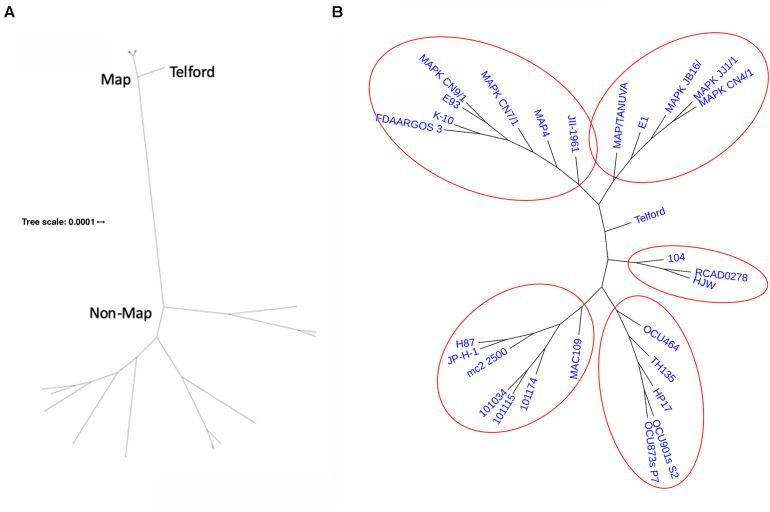
Core gene phylogeny inferred from Roary analysis of 28 *M. avium* strains with RAxML and corrected for horizontal gene transfer by ClonalFrameML. **(A)** Unrooted phylogeny visualized using iTOL with respect to the branch length distance. **(B)** Unrooted phylogeny visualized in iTOL without branch length. This enables easier visualization of the tree topology especially for the *Map* strains. *Map* type II strains appear to form 2 separated clades. Clusters are circled in red and the bootstrap value calculated with RAxML is >90%. Branch length was computed by the maximum likelihood algorithm and represents substitutions per site.

**FIGURE 9:**
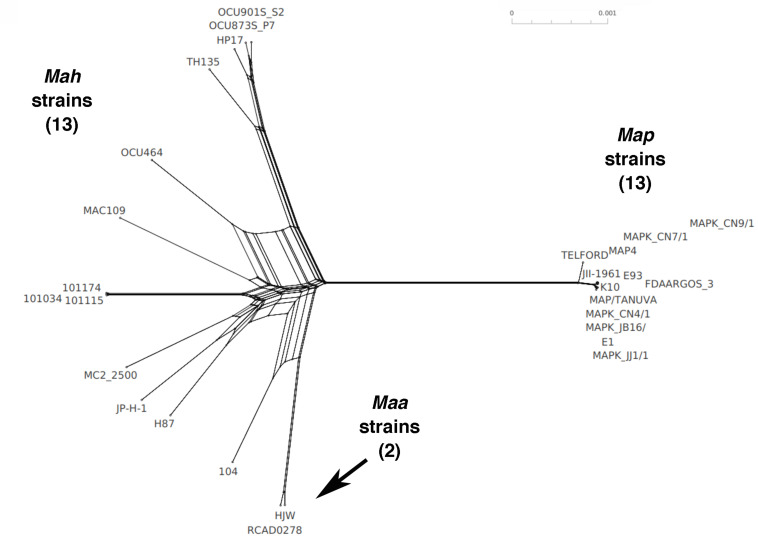
Phylogenetic network of *M. avium* subspecies. Shown is a split network phylogeny of 28 *M. avium* strains based on Roary core gene alignment obtained by Neighbor-Net analysis in SplitsTree5. Note the tight clustering of the 13 *Map* strains and the 2 *Maa* strains in contrast to the broad range of the 13 *Mah* strains.

Based on the Roary core gene analysis, a core gene SNP phylogeny was determined for all *M. avium* strains in this study. This phylogeny shows that the ovine strain Telford is well-isolated from both *Map* and non-*Map* strains (Figure 8). The tree also shows that *Mah* strain 104 is most closely related to the *Maa* strains. The *Map* type II strains separate into two main clusters similar to the *Mah* strains. Overall, the data suggest that the intermediate strains between *Mah* and *Map* are *Maa* and the *Map* type I strain. From a network phylogenetic analysis, not only do *Map* strains form a well-separated clade from other *M. avium* subspecies, but it appears some *Mah* strains are about as closely related to each other as they are to *Map* strains (Figure 9). This is supported by the many parallel lines (indicative of phylogenetic splits) on the non-*Map* side of the network. The Telford type-I strain is also clearly separated from the type-II strains of *Map*. *Mah* strains form a complex web-like tree which is in agreement with a previous study (Turenne et al., 2008) based on the diversity observed among the subspecies. Also note the *Mah* 104 strain is closely related to the two *Maa* strains. Historically, this strain was first identified as *Maa* (Mijs et al., 2002).

### Origin of Genomic Diversity in *M. avium* Subspecies

**FIGURE 10:**
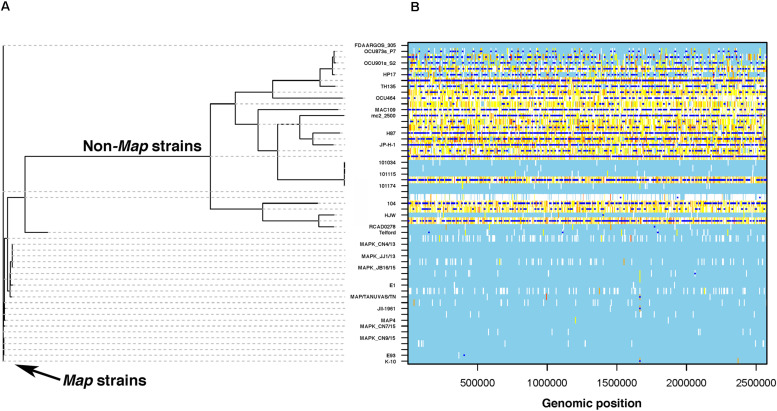
Recombination events detected in *M. avium*. ClonalFrameML was used with input from the core gene alignment by Roary and RAxML phylogeny to identify recombination induced SNPs and remove them to correct the tree. **(A)** Core gene phylogeny corrected from inferred recombination events. SNP-inducing homologous recombination due to horizontal gene transfer events in the core genome were removed to correct the true branch lengths on the tree. **(B)** Representation of recombination events along the genome. The *Y*-axis corresponds to each branch of the tree in **(A)** and the *X*-axis corresponds to the genomic position in the alignment. White vertical lines indicate nucleotide variants (SNPs) compatible with the tree, while dark blue squares represent the location of recombination sites. Note the striking number of dark blue squares in the non-*Map* strains and their lack in *Map* strains. Gray dotted lines enable easier connection between the strains shown in **(A,B)**.

From these collective results, it is clear non-*Map* strains show considerable genomic diversity, but where did this diversity originate? With the understanding that recombination can be a primary driver of diversity, ignoring this possibility when reconstructing *M. avium* phylogenies could lead to misleading conclusions about strain relationships. ClonalFrameML was used to detect recombination hot spots on a whole genome scale (Didelot and Wilson, 2015) and showed many hot spots were present in non-*Map* strains while absent in *Map* strains (Figure 10). When recombination-induced SNPs were removed to correct branch lengths, the phylogenetic relationships are more accurately reflected between *Map* and non-*Map* strains (Figure 10A). A striking number of recombination events is detected by this analysis in non-*Map* strains relative to *Map* (compare dark blue squares in Figure 10B). These events are distributed evenly across non-*Map* genomes (Figure 10B). Collectively, these data suggest that *Map* is a closed genome with horizontal gene transfer kept to a minimum.

## Discussion

To increase understanding of *M. avium* genome organization, only completely sequenced *M. avium* subspecies genomes were considered. Insufficient numbers of complete genomes had been a limitation in earlier studies, but recently a similar number of *Map* and non-*Map M. avium* genomes have emerged. Overall, we show that among the three *M. avium* subspecies studied, *Map* is a relatively stable, closed genome while *Mah* and *Maa* show more horizontal gene transfer and accessory genome components. *Map* clearly has a very homogenous genome, except for the Telford type I strain which has a different genomic organization and an order of magnitude more SNPs compared to the 12 type II *Map* strains. It will be interesting to examine additional type I and type III strains of *Map* once complete genomes become available. Currently, there are two draft genomes available for the type III strain of *Map* (Bannantine et al., 2012; Mobius et al., 2015), but no complete genome sequences. We recognize that analyzing 12 of 13 type II genomes may skew the data to enable *Map* to appear more homogeneous, but that was what met the criteria for inclusion in this study and highlights the current focus of research on these strains. Other studies using SNP-based analyses of selected genes or VNTRs of incomplete genomes suggest ovine strains are more polymorphic than type II bovine strains (Turenne et al., 2008; Bryant et al., 2016; Sohal et al., 2019). Furthermore, *Map* genomes aligned at a common start revealed that type II strains have a highly homologous genome synteny while the sheep strain showed large rearrangements.

Conversely, the non-*Map* members of *M. avium* have genomes that are mosaic in nature similar to what has been described elsewhere (Turenne et al., 2008; Uchiya et al., 2017; Yano et al., 2017). The homogeneous *Map* and heterogeneous non-*Map* genomes are well illustrated by the following observations. *Map* was divided into two hsp65 sequevars, while non-*Map* binned into 6 sequevars. The total numbers of SNPs were 10 times more in non-*Map* than *Map* and the non-synonymous SNPs were consistently higher than synonymous SNPs in *Map* genomes while the opposite is true for non-*Map*. The resulting phylogenies showed distinct clustering between *Map* and non-*Map*. Plasmid DNAs are present in some non-*Map* strains, but not in any *Map* strains. Inferred recombination sites are significantly more prevalent in non-*Map* genomes. And finally, there is a large accessory genome in non-*Map* strains. Collectively, this further demonstrates the heterogeneous nature of *Mah* genomes and the conserved nature of *Map* genomes. One potential reason *Map* genomes appear very stable with low numbers of SNPs and lack of rearrangements could be due to minimal selective or environmental pressure that stems from the passive lifestyle niche *Map* exists in. An alternative explanation may be that *Map* has spread worldwide relatively recently through livestock transportation in the industrialized world.

It is interesting to note the strong bias toward two of the four *M. avium* subspecies in terms of genomes sequenced. From this set of 28 genomes, 26 comprise either *hominissuis* or *paratuberculosis*. This does not include yet another *Mah* MAH11 sequence recently completed (Dragset et al., 2019). This bias is most likely due to the fact that *Map* is a significant veterinary pathogen and *M. avium* subspecies *hominissuis* is a primary cause of human lung infections and lymphadenitis in pigs. Conversely, *silvaticum* and *avium* subspecies are either bird pathogens or environmental commensals and hence there is little funding or effort dedicated to understanding their genetic makeup. There is another possibility for the lack of representation of the *silvaticum* subspecies in sequence repositories. *M. avium* subspecies *silvaticum* was originally proposed by Thorel and coworkers based on specific growth conditions (Thorel et al., 1990), however, it is likely not a separate subspecies. Phylogenetic evidence and other analyses suggest that the *silvaticum* subspecies groups very closely to the *avium* subspecies (Turenne et al., 2006; Bryant et al., 2016; Tortoli et al., 2019). Also, there is very little sequence in public databases and what is present clusters tightly to *Maa*. Only the *silvaticum* type strain, designated ATCC 49884, has been carried over in the analyses of many phylogenetic studies as no others are available and there may be only one other isolate circulating with no new isolates having been reported or sequenced. Therefore, it is our position that the only *M. avium* subspecies that should be recognized are *avium*, *hominissuis* and *paratuberculosis*, however, very recent data obtained by calculating ANI and genome-to-genome distance suggest the addition of a new subspecies termed *M. avium* subspecies *lepraemurium* (Tortoli et al., 2019). This new subspecies differs from subspecies *avium* by 4 bp in the 16S rRNA gene.

One area where annotations have improved dramatically has been in GC-rich organisms like that observed in *M. avium* genomes. The new RefSeq annotation of these genomes has enabled the standardization and identification of pseudogenes. Also, the number of hypothetical proteins has dropped significantly with the new annotation. The resequenced K-10 with updated annotation (Wynne et al., 2010) had 3,100 hypothetical proteins, compared to the RefSeq annotation, which has only 793. All *M. avium* genomes in this study are now below 20% hypothetical proteins (Table 3). Despite the number of complete genomes for *M. avium* and their RefSeq annotation, there are still a considerable number of hypothetical proteins. These should become the target of future studies examining their function and a prioritized list, which includes proteins with a predictable biochemical activity, has already been developed (Galperin and Koonin, 2004). Interestingly, a high concentration of hypothetical proteins (67%) in the K-10 strain are listed among RefSeq annotated genes that were not in the initial annotation (Supplementary Table S2). Many of these newly predicted genes (82%) are also core genes in *Map*, increasing the interest to study these further.

Even with these complete genome sequences now available and a phylogenetic network established, a progenitor strain cannot be unequivocally determined from these analyses. The larger average genome size for *Mah* makes it tempting to suggest it is the progenitor, but there is no clear evidence for this hypothesis. It could simply be a more open genome that readily takes up new genes through recombination. Overall, *M. avium* has evolved through well-described pathways which include insertions/deletions, recombination and modifications such as SNPs. A model can be proposed once the order of these documented genomic modifications becomes known.

Whole genome similarity analysis based on nucleotide level comparisons such as ANI and Jaccard similarity are ideally performed on complete genomes sequences to obtain static, reproducible results. The *M. avium* strains in this study are closely related and belong together at the subspecies level as shown by > 98% ANI values, which drop quickly when comparing sequences outside *M. avium*, even with *M. intracellulare*, which is a member of the MAC. The Jaccard pairwise similarity values showed better discrimination of *M. avium* strain relatedness. The Jaccard method has not been widely adapted because detailed genome annotations were lacking, however, with RefSeq annotations available, similarity measurements using the Jaccard coefficient provide a more relevant and simplified basis for genome comparison (Jay et al., 2012). At the gene level, *Map* and *Mah* form well-separated clusters with *Maa* and the *Map* ovine strain as intermediates between these principle clusters (Figures 8, 9).

The two Egyptian *Map* strains E1 and E93 do not have complete copies of the hallmark IS900 insertion sequence (Table 3). We identified 17 incomplete copies of IS900 represented in 34 fragments of ∼200 bp at each extremity of the IS element. Furthermore, both of their genome sizes are smaller than for all other *Map* strains (Table 3), which is also detectable in the Mauve alignment (Figure 4). We confirmed that these two genomes are type II strains on the basis of SNPs in the *gyrA* and *gyrB* genes (Castellanos et al., 2007) and they also contain the LSP20 region as do all other type II strains (Semret et al., 2004). These two genomes were not *de novo* assembled, but were assembled using K-10 as the reference and not all reads mapped to the reference (Amin et al., 2015). For these reasons, we suspect a mistake may have been introduced in the assembly of those genomes where the repeat elements might have been masked out during an initial assembly, but then not added back into the final assembly. All other type II strains in this study had either 16 or 17 copies of IS900.

Surprisingly, only 16 SNPs were detected in FDAARGOS_305, which suggests it is essentially the same as K-10. These two genomes also clustered tightly by core gene phylogeny (Figure 8B). The FDAAROGOS_305 strain is part of a much larger microbial genome sequencing effort that began in May of 2014 and is funded by two US government agencies, the Department of Defense and the Food and Drug Administration. Termed FDA-ARGOS, this large sequencing effort currently has over 1,000 microbial genomes sequenced (Sichtig et al., 2019). When researching the source of the FDAAROGOS_305 strain, we discovered that FDA-ARGOS re-sequenced the K-10 strain that was deposited in the ATCC culture collection. This explained why the two genomes were separated by less than 20 SNPs and showed no major genomic rearrangements.

The core genome represents genes that are present in all strains while the pangenome encompasses all genes, orthologous or not, among the species. In *E. coli* for example, only 39% of the genes comprise the core genome and genome sizes among *E. coli* strains can vary by more than 500 kilobase pairs (Touchon et al., 2009). These numbers are similar for non-*Map M. avium* strains. By contrast in *Map*, the core genome is 80% (Figure 5) with genome sizes varying by only 126,426 bp among the 13 genomes analyzed. This fact, combined with recombination analysis (Figure 10), suggests that *Map* genomes do not show evidence of widespread horizontal gene transfer and that one strain can epitomize the *Map* subspecies, at least for type II strains. Previous studies have shown that *Mah* is more like *E. coli* in that it rapidly acquires new genes, contains plasmids, and the gene repertoire is larger than *Map* (Yano et al., 2017). Thus, no single *Mah* strain could adequately represent this subspecies.

The *M. avium* genomes that have been sequenced, along with others in progress, will serve as the foundation for population genomics and evolution of the MAC. These data have narrowed the gap between population genetic and phylogenetic approaches to study genome evolution, both of which are important to understanding the effect of gene gain and loss on adaptation and genome synteny. We have observed that the core genome evolves primary through SNPs, and possible recombinant events, while the accessory genome is acquired through horizontal gene transfer. Finally, the *Map* genome is very closed and stable, which is not the case for non-*Map* genomes.

## Data Availability Statement

The datasets generated for this study can be found in the EVA, Project: PRJEB39090 and Analyses: ERZ1461588.

## Author Contributions

JB conceived and designed the study. All authors made substantial contributions to the analysis and writing of the manuscript.

## Conflict of Interest

The authors declare that the research was conducted in the absence of any commercial or financial relationships that could be construed as a potential conflict of interest.

## Footnotes

1 https://github.com/vcflib/vcflib

2 https://github.com/Papos92/MISTReSS

## Supplementary Material

FIGURE S1 Mauve alignment between *Map* type II strains without prior alignment of dnaA as the starting point. The alignment is organized into one horizontal section per input genome sequence. Each genome track contains the accession number and strain of the genome sequence along with a base pair scale showing the sequence coordinates for that genome. Blocks of various colors above and beneath the center line represent defined genome regions. Similar colored blocks in different genomes represent regions that aligned in each genome. When a block lies above the center line the aligned region is in the forward orientation relative to the K-10 genome sequence (top). Blocks below the center line indicate regions that align in the reverse complement (inverse) orientation. Comparison of this figure with Figure 4 in the manuscript shows a striking difference.

FIGURE S2 Pangenome map of all 28 *M. avium* strains. The outer circle shows the variable regions in dark gray, while the core regions are a lighter shade of gray. The core genes on the positive strain are shown in the middle ring and on the negative strand are shown in the inner ring. Those core genes are color coded with the legend. This image was generated in PanOCT.

TABLE S1 Correlation of old and new locus tags for *Map* K-10. Shown is a complete list of the RefSeq locus tags for *Map* K-10 along with the corresponding old locus tags which have been used in the scientific literature. In addition, the protein ID and description are given for coding sequences. The “NA” designation in the old locus tag column indicates a gene that was not included in the original annotation. If “NA” is present in both the old locus tag and Protein ID columns, that gene is a pseudogene.

TABLE S2 RefSeq annotated genes not present in the initial *Map* K-10 annotation. Shown are the cds and gene numbers as well as the RefSeq locus tag, protein ID and expression product.

TABLE S3 Pseudogenes in *Map* K-10. A total of 199 pseudogenes are listed along with their start and stop coordinates on the K-10 RefSeq annotation. The old locus tag is listed if applicable.

TABLE S4 Complete and partial copies of IS1245 among non-*Map* genomes. The criteria to be listed as a functional copy of IS1245 is that the product accession must be WP_011725525.1 and the size of the element must be 1,233 bp. Any other size was considered a partial copy and a pseudogene. Each IS1245 copy is listed along with strain designation and locus tag.

TABLE S5 Single nucleotide variant in MAP_RS05220 (MAP1025) distinguishes *Map* and non-*Map* strains. All the strains from this study are listed along with the SNP at position 83 within the MAP_RS05220 gene and the RefSeq coordinate.

TABLE S6 *hsp65* sequevars among *M. avium* strains. Strain 104 was used as the reference in this analysis similar to Turenne et al., 2006. Nucleotides are shown for all positions within *hsp65* for *Mah* 104 and those strains with the same nucleotide as *Mah* 104 are marked by a dash. Nucleotides that differ from *Mah* 104 are indicated by listing the divergent nucleotide. Nucleotide positions highlighted in red are SNPs not reported by Turenne et al., 2006.

TABLE S7 Core genes in *Map* as determined by Roary analysis.

TABLE S8 Core genes in non-*Map* as determined by Roary analysis.

TABLE S9 *Map* core genes present by PanOCT analysis.
