# Supplementary figures and images for: Genetic Diversity Among Mycobacterium avium Subspecies Revealed by Analysis of Complete Genome Sequences
Source: Front Microbiol. 2020 Aug 7;11:1701. doi: 10.3389/fmicb.2020.01701 (PMC7426613; doi:10.3389/fmicb.2020.01701)

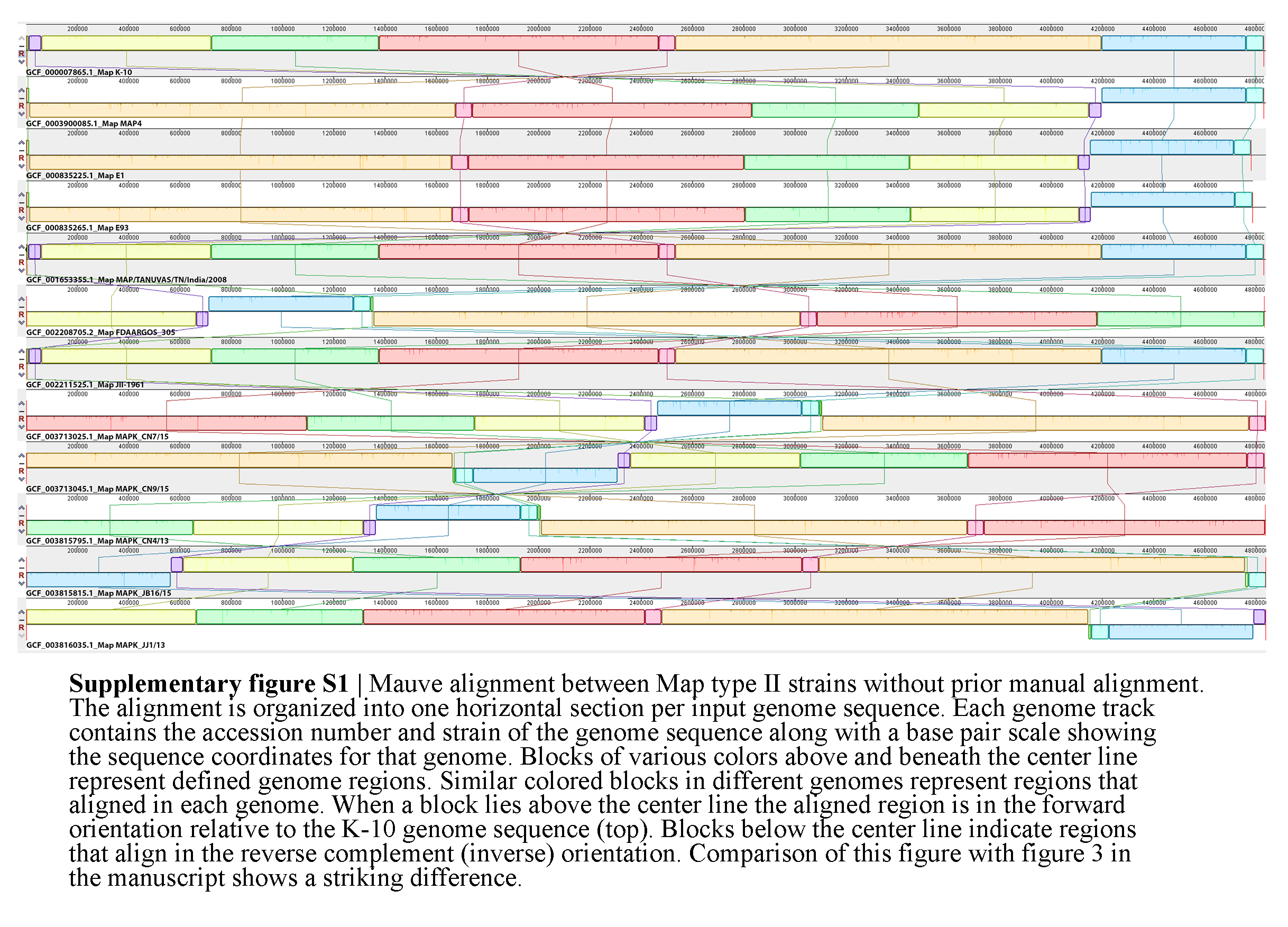

Supplement: FIGURE S1 — Mauve alignment between Map type II strains without prior alignment of dnaA as the starting point. The alignment is organized into one horizontal section per input genome sequence. Each genome track contains the accession number and strain of the genome sequence along with a base pair scale showing the sequence coordinates for that genome. Blocks of various colors above and beneath the center line represent defined genome regions. Similar colored blocks in different genomes represent regions that aligned in each genome. When a block lies above the center line the aligned region is in the forward orientation relative to the K-10 genome sequence (top). Blocks below the center line indicate regions that align in the reverse complement (inverse) orientation. Comparison of this figure with Figure 4 in the manuscript shows a striking difference. [file Image_1.TIF]

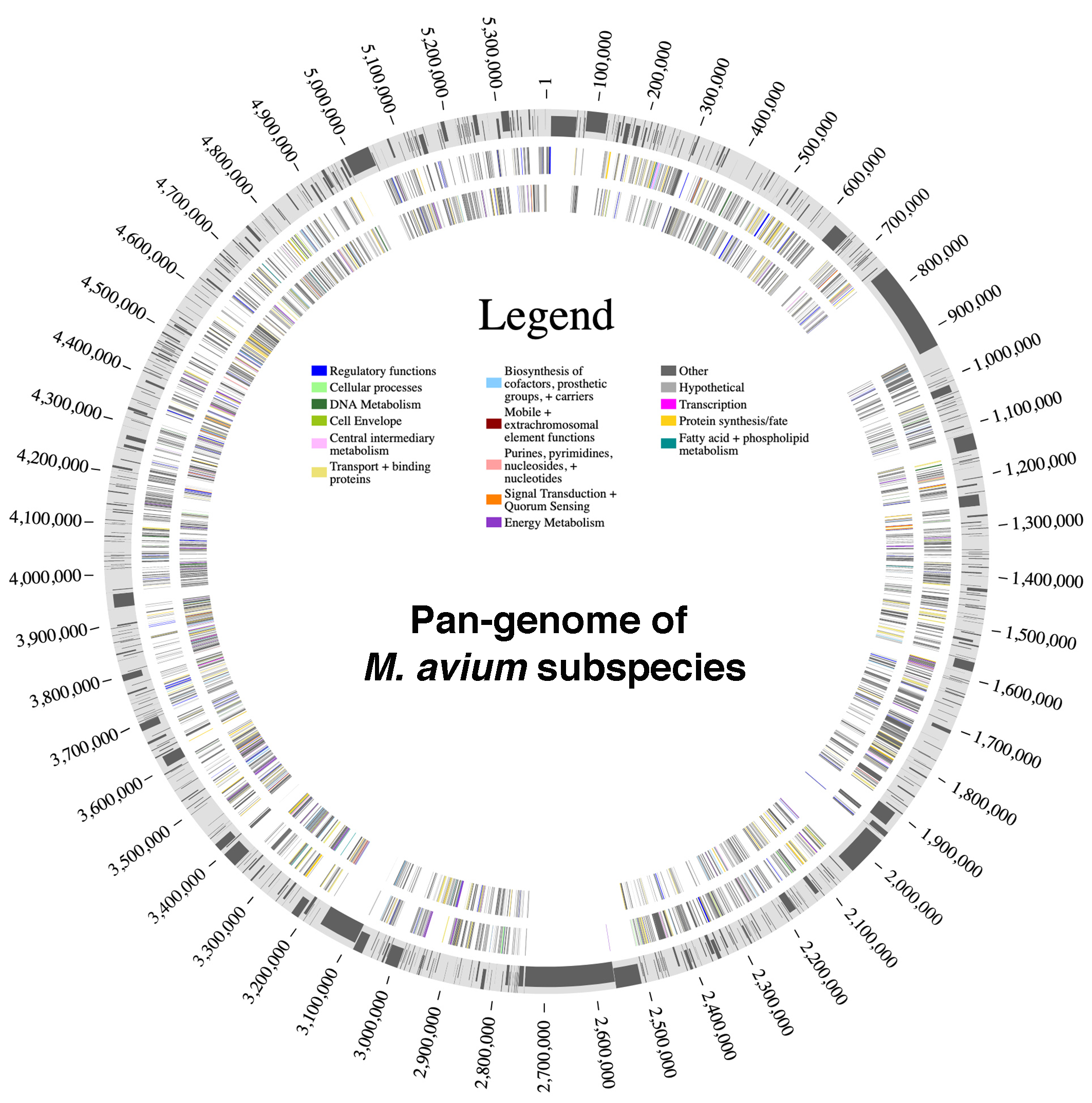

Supplement: FIGURE S2 — Pangenome map of all 28 M. avium strains. The outer circle shows the variable regions in dark gray, while the core regions are a lighter shade of gray. The core genes on the positive strain are shown in the middle ring and on the negative strand are shown in the inner ring. Those core genes are color coded with the legend. This image was generated in PanOCT. [file Image_2.TIF]
